# Engaging veteran stakeholders to identify patient‐centred research priorities for optimizing implementation of lung cancer screening

**DOI:** 10.1111/hex.13401

**Published:** 2021-12-10

**Authors:** Alice Yan, Katinka Hooyer, Onur Asan, Mark Flower, Jeff Whittle

**Affiliations:** ^1^ Center for Advancing Population Science, Division of General Internal Medicine, Department of Medicine Medical College of Wisconsin Wauwatosa Wisconsin USA; ^2^ Department of Family and Community Medicine Center for Healthy Communities and Research, Medical College of Wisconsin Wauwatosa Wisconsin USA; ^3^ School of Systems & Enterprises Stevens Institute of Technology Hoboken New Jersey USA; ^4^ Department of Psychiatry and Behavioral Medicine Medical College of Wisconsin Milwaukee Wisconsin USA; ^5^ Department of Medicine Clement J. Zablocki VA Medical Center Milwaukee Wisconsin USA

**Keywords:** citizen science, diagnostic imaging, early cancer detection, lung cancer, patient participation, Veterans

## Abstract

**Background:**

Patient engagement in research agenda setting is increasingly being seen as a strategy to improve the responsiveness of healthcare to patient priorities. Implementation of low‐dose computed tomography (LDCT) screening for lung cancer is suboptimal, suggesting that research is needed.

**Objectives:**

This study aimed to describe an approach by which a Veteran patient group worked with other stakeholders to develop a research agenda for LDCT screening and to describe the research questions that they prioritized.

**Methods:**

We worked with Veterans organizations to identify 12 Veterans or family members at risk for or having experience with lung cancer to form a Patient Advisory Council (PAC). The PAC met repeatedly from June 2018 to December 2020, both independently and jointly, with stakeholders representing clinicians, health administrators and researchers to identify relevant research topics. The PAC prioritized these topics and then identified questions within these areas where research was needed using an iterative process. Finally, they ranked the importance of obtaining answers to these questions.

**Results:**

PAC members valued the co‐learning generated by interactions with stakeholders, but emphasized the importance of facilitation to avoid stakeholders dominating the discussion. The PAC prioritized three broad research areas—(1) the impact of insurance on uptake of LDCT; (2) how best to inform Veterans about LDCT; and (3) follow‐up and impact of screening results. Using these areas as guides, PAC members identified 20 specific questions, ranking as most important (1) innovative outreach methods, (2) the impact of screening on psychological health, and (3) the impact of outsourcing scans from VA to non‐VA providers on completion of recommended follow‐up of screening results. The latter two were not identified as high priority by the stakeholder group.

**Conclusions:**

We present an approach that facilitates co‐learning between Veteran patients and providers, researchers and health system administrators to increase patient confidence in their ability to contribute important information to a research agenda. The research questions prioritized by the Veterans who participated in this project illustrate that for this new screening technology, patients are concerned about the practical details of implementation (e.g., follow‐up) and the technology's impact on quality of life.

**Patient or Public Contribution:**

Veterans and Veteran advocates contributed to our research team throughout the entire research process, including conceiving and co‐authoring this manuscript.

## 1 INTRODUCTION

Research funds are limited, making it important to use them in ways that maximize impact on patients and providers. Historically, research priority setting has been controlled by scientists^1^, ^2^ rather than individuals affected by the conditions being studied.^3^, ^4^ Increasingly, patient engagement is seen as a strategy to ensure that research leads to effective and responsive healthcare service delivery.^5^, ^6^, ^7^ Thus, those developing research priorities have begun to seek out the voice of affected individuals in developing research agendas.^1^, ^8^ There is some evidence that involving patients throughout the research process, starting with setting the research agenda, helps ensure that the research conducted is patient‐centred,^9^ while without patient representation, research agendas may not align with information needs of greatest importance to patients.^10^

One area where patient input has been limited is lung cancer screening research. The National Lung Screening Trial (NLST) and other studies have demonstrated that screening with low‐dose computed tomography (LDCT) reduces lung cancer mortality among adults at high risk of lung cancer.^11^, ^12^ Consequently, the US Preventive Services Task Force now recommends lung cancer screening for such individuals.^13^ However, these screenings cause harms (anxiety, unnecessary surgeries, radiation exposure, etc.) as well as benefits (decreased lung cancer mortality) for patients. Research suggests that decisions regarding lung cancer screening are difficult for many patients.^14^, ^15^ It is thus not surprising that uptake has been slow and uneven, threatening to worsen existing disparities in lung cancer outcomes.^16^, ^17^ Consequently, there is considerable interest in research regarding how to optimize LDCT implementation to maximize population benefit.^18^, ^19^, ^20^

Lung cancer is of particular importance to US military Veterans as they are often at increased risk for lung cancer due to high rates of smoking and exposure to carcinogens (e.g., Agent Orange). Higher rates of lung cancer have been observed in Gulf War Veterans^21^ and among Marine ground troops who served in Vietnam.^22^ Not only is their incidence of this cancer higher but their survival rates are also lower than among civilian populations.^23^ With three‐fourths of the nearly 22 million Veterans receiving some or all of their medical treatment outside the Veterans Administration (VA),^24^ engaging Veterans in research agenda setting for LDCT implementation can help guide research both within and outside VA.

In this paper, we describe an approach by which a Veteran patient group developed a research agenda for LDCT screening in collaboration with other stakeholders. We then present the patient‐centred research priorities around optimizing LDCT implementation; this was generated through this collaboration.

## 2 METHODS

### 2.1 Study design

**Figure 1:**
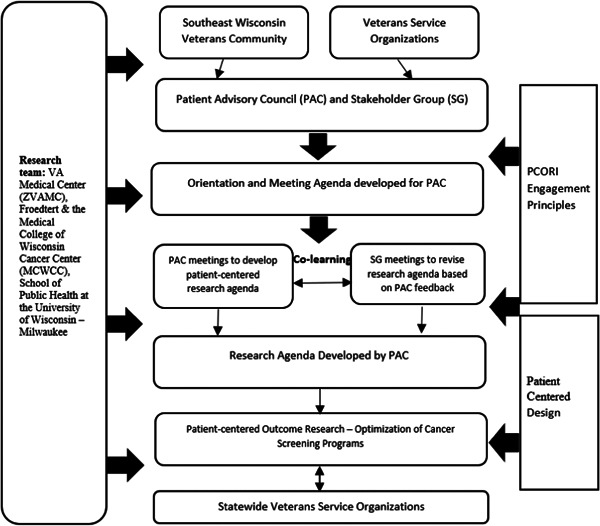
Project organizational structure and the design

This study reports an approach for identifying patient‐centred research questions around optimizing LDCT implementation through facilitating a process of two‐way co‐learning between two groups of stakeholders: the Patient Advisory Council (PAC) and Stakeholder Group (SG). Co‐learning is one of the Patient‐Centered Outcome Research Institute (PCORI) principles of patient and stakeholder engagement. The project focused on Southeast Wisconsin, where the research team has deep connections to Veteran groups. Figure 1 shows the project's organizational structure and design, which is consistent with PCORI engagement principles,^25^ including (1) reciprocal relationships, (2) co‐learning, (3) partnership and (4) trust, transparency and honesty.^25^, ^26^ This structure is designed to facilitate (1) a collaborative relationship between Veteran patients and other stakeholders; (2) co‐learning where researchers and clinicians learn about patient priorities and patients become engaged as partners in the research process; and (3) transparency and trust between groups with different relationships to research. Through a series of co‐learning meetings within and between the PAC and SG groups, a research agenda was developed, which can inform Patient‐Centered Outcomes Research directed at optimization of lung cancer screening programmes. Based on having been involved throughout the process, statewide Veterans Organizations are now well positioned to both consume this type of research and provide ongoing input as the body of research evolves.

### 2.2 Recruitment

We recruited 12 PAC members from various racial backgrounds based on (1) fitting the NLST entry criteria (age 55–74 years, 30 or more pack‐years of cigarette smoking history and smoking within the previous 15 years),^13^, ^27^ (2) perceive themselves as being high risk for lung cancer, but not eligible for the NLST, or (3) having a personal or family ‘lived experience’ with lung cancer. Only Veterans or their family members were included. Although two members were not Veterans (they were family members of Veterans); we will refer to this group as ‘Veterans’ throughout the manuscript. We recruited PAC members through direct outreach to individuals known to our Veteran community partners, placement of electronic and printed recruitment materials in prominent locations within the community and word‐of‐mouth referrals from Veterans we approached about participation.

We recruited 10 SG members to represent lung cancer providers (VA and academic radiologists, thoracic surgeons and pulmonologists), researchers, health system administrators and patient advocates. Our patient advocates included senior Wisconsin members of the American Cancer Society, American Legion and Vietnam Veterans of America organizations. Their involvement was designed to both facilitate dissemination of project products back to the Veteran community and to include a Veteran voice in discussions by the SG. These Veterans were not involved in PAC activities.

PAC and SG members were paid based on meeting attendance in accordance with PCORI's best practice.^28^ Some SG members were unable to accept payment as VA employees.

### 2.3 Meeting principles

Meetings were based on an evidence‐based design called the ‘building block approach’ for consensus building.^29^ This model suggests that groups have time to discuss separately and independently when ‘potential conflicting issues’ arise. As PAC members have the potential to defer to the SG as ‘experts’, and the SG may expect this deference, the PAC and SG met separately for initial meetings. To develop the PAC's confidence in their value to the research team, they were asked to review and provide feedback on research tools such as recruitment strategies, data collection instruments and outreach letters. After each PAC meeting, we emphasized how their experience provided perspectives that the research team could not have acquired in any other way through a summary of ‘How the PAC is making a difference’ based on input that they had provided during that meeting. We also coached the SG members regarding the importance of gaining Veteran perspectives and warned them against dominating interactions. All PAC meetings occurred in person in a reserved VA conference room, except the last, which was virtual due to COVID‐19.

We arranged two joint meetings for PAC and SG members in year two, adhering to the PCORI engagement principle of co‐learning. During the meetings, we used moderation techniques to ensure that there were opportunities for both groups to speak. Despite these efforts, after each joint meeting, we discussed how to improve the dialogue with both groups (PAC and SG), so that our moderation strategy evolved over time. Additionally, PAC feedback caused us to orient PAC members more fully about the planned joint meeting activities before that meeting. A designated staff took minutes for all meetings. The project staff also summarized each meeting immediately after the meetings and sent the meeting recap follow‐up emails to all PAC and SG members.

#### 2.3.1 PAC and SG meetings

PAC members met quarterly, and each meeting lasted approximately 2 h, with lunch provided. Between June 2018 and December 2020, the PAC had seven meetings: one kick‐off, two joint meetings with SG and five PAC meetings. PAC meetings had two major objectives: (1) share personal experiences with lung cancer screening, diagnosis and/or treatment and (2) develop a Veteran‐centred research agenda in collaboration with the SG. Responding to PAC requests, learning sessions to address questions related to LDCT from the patients' perspective were incorporated into meetings.

The SG met less regularly, and their meetings were scheduled to coordinate with PAC needs. The SG met three times per year (the last meeting was cancelled due to COVID‐19) for a total of five meetings, including one kick‐off, two joint meetings with PAC and two SG meetings. The main objectives of the SG meetings were to: (1) translate the PAC's ideas into research questions and (2) generate a sustainability plan to maintain the PAC for future research engagement.

#### 2.3.2 PAC and SG joint meetings

The PAC and SG convened as a single group two times (joint meetings). These learning collaborative 2‐h sessions were designed for co‐learning. The third joint meeting, a project celebration, was cancelled due to the COVID‐19 pandemic. The goals of the first two joint meetings were to develop a research agenda and identify research questions. The project team had a synthesis responsibility during the ‘reaching consensus’ section on the research agenda, combining the agendas developed by the two groups. The two sets of agendas were revised and negotiated at each joint meeting and presented back to members at the individual meetings (of SG and PAC) in between those two joint meetings.

#### 2.3.3 Research agenda development process

**Figure 2:**
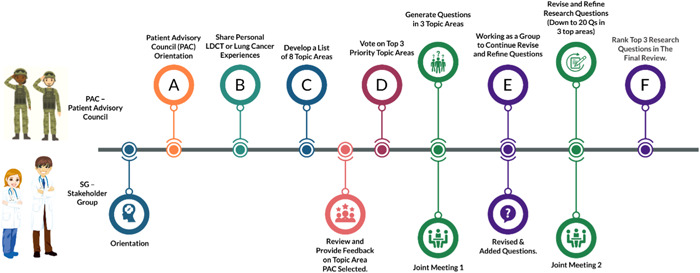
PAC and SG meetings and research agenda development workflow. PAC, Patient Advisory Council; SG, Stakeholder Group

The research agenda development process involved several steps, including PAC and SG independent meetings, PAC and SG joint meetings and a final voting. The voting process was anonymous following the REPRISE priority setting reporting checklist formulated by Tong et al.^30^ Figure 2 shows the sequence of PAC and SG meetings and the workflow of developing the research agenda as a result of these meetings. First, PAC developed a list of eight topic areas that emerged during key discussions around personal experiences in early PAC meetings. Second, PAC members voted on their top three priorities, from among these topic areas (Table 1). The top three topic areas were as follows: (1) ‘How does insurance impact lung cancer screening?’ (2) ‘What is the best way to do outreach to Veterans for screening?’ (3) ‘What happens after people get screened?’ Third, in the first joint meeting of the PAC and SG, questions were generated under each topic area. After the joint meeting, the project team (the PIs and project staff) edited the list of questions for clarity. The PAC and SG discussed these questions during independent meetings, and then the project team shared the topics discussed across the groups. Fourth, during the second joint meeting, the PAC and SG continued to revise, refine or add questions. This led to 20 questions, which the project team again edited for clarity. Finally, the PAC ranked their top three priority research questions in the final review. Table 2 shows the final PAC‐approved agenda with the top three research questions in bold (Table 2). These research questions were as follows: (1) What outreach approach or combination of outreach approaches (e.g., social media, mailers, health fairs) yield the best outcome? (2) Do screening results affect psychological health? (3) Does the rate of follow‐up and completing scans 2 and 3 differ if the initial scan is performed within VA hospitals versus outsourced? This approach aligns with PCORI's standard process for prioritizing patient‐centred research questions, which asks stakeholders to judge topics based in part on the topic's importance to themselves.^31^

**Table 1:** Top three prioritized research topic areas (*n* = 9)

Top 3 topic areas	# of votes	Rationale
1. How does insurance impact lung cancer screening?	8	The cost of follow‐up care and the cost to Vets who are not service connected/seeking care at VA are barriers. Years of military service impacts co‐pay.
2. What is the best way to do outreach to Vets for screening?	5	Vets have varying experiences with the military, served in different eras, respond to different kinds of messaging and use different types of media.
3. What happens after people get screened?	9	Questions were raised on the stress that screening created for Vets, how results were conveyed and their psychological impact and whether Vets followed up after screening.

**Table 2:** Research questions and PAC members' vote counts (*n* = 8)

Questions		Numbers of votes as #1	numbers of votes as #2	numbers of votes as #3	Sum
*Best way to do outreach to veterans for screening*					
1	Could lung cancer screening navigators increase the number of veterans who receive appropriate LDCT?	1	0	0	1
2	**What types of outreach or combination of outreach (e.g., social media, mailers, health fairs) yield the best outcome?**	**3**	**1**	**1**	**5**
3	How do we reach the 70% who do not go to the VA, but are eligible?	0	0	0	0
4	Should media messaging (communications) vary for different Veterans? For example, is it better to simply tell Veterans 'time for your LDCT' or is it better to provide a lot of information about the pros and cons of LDCT?	0	1	0	1
*What happens after people get screened?*					
5	Do screening results affect smoking? (does it push them to stop?)	0	0	1	1
6	**Do screening results affect psychological health?**	**3**	**1**	**0**	**4**
7	What factors influence patients' decisions to follow up or not to follow up on abnormal screening results?	0	1	1	2
8	Does the way in which results are conveyed to patients affect their response?	1	0	0	1
9	Was the screening and follow‐up a positive or negative experience?	0	0	0	0
*How does having health insurance affect lung cancer screening?*					
10	Does the Mission Act increase the number of Veterans receiving LDCT?	0	0	2	2
11	Do Veterans who are not VA covered get screening?	0	0	0	0
12	For people receiving LDCT outside of the VA, is the follow‐up as reliable/positive (in terms of patient experience) as if it was ordered within the VA?	0	0	0	0
*Other*					
13	Do more years of screening for an individual improve outcomes? Note that currently, CMS pays for 3 scans done (at least) every 12 months.	0	0	1	1
14	Do patient characteristics affect LDCT use?	0	0	0	0
15	Do patient characteristics affect LDCT outcomes?	0	1	0	1
16	Do results differ between scans (of VA users) performed within VA versus outsourced?	0	0	1	1
17	**Does the rate of follow‐up and completing scans 2 and 3 differ if the initial scan is performed within VA hospitals versus outsourced?**	**0**	**2**	**1**	**3**
18	Is there a threshold for conducting accurate screens? (e.g., two people conducting 200 scans or 17 conducting 20 scans). It was noted that this type of work may have been completed: certainly CMS requires documentation of volume in their QI programme, which is linked to payment.	0	0	0	0
19	Does the rate of follow‐up and completing scans 2 and 3 compare among persons receiving results remotely (e.g., telephone, patient portal, etc.) versus in person versus using a shared decision‐making tool?	0	1	0	1
20	Do false‐positive rates vary regionally? For example, in VA, do certain VISNs have a higher percentage of screening?	0	0	0	0
	**Total**				**24**

Abbreviations: LDCT, low‐dose computed tomography; PAC, Patient Advisory Council.

## 3 RESULTS

### 3.1 Characteristics of PAC and SG members

Among 12 PAC members we recruited, one‐third were women, one was African American and one was Hispanic. The 10 SG members included 3 VA and 3 MCW healthcare providers, an MCW population health scientist and representatives from the American Legion, the Vietnam Veterans of America (VVA) and the American Cancer Society.

### 3.2 Research agenda

The agenda reflects Veterans' prioritized research topics influenced by their life experiences. These topics were different from and sometimes viewed as less significant than the topics identified by researchers.

#### 3.2.1 Priority topics

The PAC prioritized their top three topics of interest by their third meeting (Table 1). The first topic was the impact of insurance coverage on lung cancer screening. Members acknowledged that not all Veterans have access to VA health benefits or want to seek care at VA hospitals. Veteran Benefit Administration ratings influence whether a Veteran can access VA healthcare and can impact screening, diagnosis and treatment. Additionally, health insurance is complex, co‐pays are constantly changing and policies use technical language, making it difficult to understand benefits. This can be overwhelming, especially for Veterans who may contend with mental health or physical challenges. PAC members felt that addressing health insurance was important because ‘…at what point do people make decisions, not on money but other factors?’ PAC noted that research evidencing that Vets get cancer at a higher rate than the civilian population might get Congress to allocate more money for Veterans, so that more early screening could be conducted.

The second topic focused on the best way to do outreach to encourage screening. Much of the concern on outreach revolved around those Veterans who were ‘falling through the cracks’: the young, uninsured, isolated and marginalized (e.g., dishonourably discharged or rural Veterans). Notably, PAC members provided many strategies for doing outreach, with attention to Veterans not seeking VA care. This included peer mentoring, word‐of‐mouth, social media and tapping into community organizations that sponsor Veteran activities.

The third priority topic focused on what happens after people get screened. Concerns were rooted in the psychological and emotional impact stemming from waiting for results, the uncertainty of the next steps and potential financial distress. PAC pointed to how results that are conveyed to patients could affect how they respond (i.e., by mail, phone call or clinical visit). Major concern was expressed over whether Veterans followed up after a positive screen, how to mitigate denial and the impact of military training on self‐care (e.g., values of selfless service and putting the collective [family, work] first).

#### 3.2.2 Research agenda and priority questions

In the three prioritized topic areas, two cycles of feedback generated 20 research questions (Table 2). A few questions posed by the SG were included, although they fell outside of the PAC's priority topics (e.g., Do false‐positive rates vary regionally?). PAC prioritized the following three questions: (1) What types of outreach or combinations of outreach (e.g., social media, mailers, health fairs) yield the best outcome? (2) Do screening results affect psychological health? (3) Does the rate of follow‐up and completing scans 2 and 3 differ if the initial scan is performed within VA hospitals versus outsourced? Notably, no questions generated under the third prioritized topic about health insurance (‘How does insurance impact lung cancer screening?’) received votes.

(1) What Outreach Provides the Best Outcome?
  Rather than generating questions in this area, PAC members focused on developing strategies (e.g., social media aimed at younger Veterans) that might positively impact screening behaviour. Most members felt that raising awareness of and promoting screening was important for improving Veteran health outcomes. They suggested that researchers could help identify and test outreach approaches that would work for different Veterans. This was important for health topics that do not come up in clinical appointments and could be missed. They offered guidance such as noting that Veterans have varying backgrounds and live in different locations, and so likely require different ways of engaging. They warned that partial information could scare Veterans and lead to permanent avoidance. Awareness of lung cancer risk by younger Veterans was a particular concern.
(2) Do screening results affect psychological health?
  This was a prioritized topic early on in PAC meetings and remained so through multiple iterations of the research agenda. Most PAC members expressed concern over waiting: waiting to see a specialist, waiting for results and waiting for further care. The emotional burden and stress of uncertainty were considered potential triggers for Veterans who are ‘already psychologically fragile’. The stress of waiting for results or having to make a decision based on complex health information can compound mental health issues. Some PAC members felt that this added anxiety might be a cause for screening avoidance.
(3) Do rates of follow‐up differ if the initial scan is performed within versus outside the VA?

PAC discussed the varying opinions regarding the quality of Veteran‐specific patient‐centred care outside VA. If Veterans are falling through the cracks and missing life‐saving follow‐up, this needs to be identified and interventions need to be developed. This question was not related to insurance coverage; rather, it reflected Veterans' concern over civilian clinicians' understanding of military culture and awareness of Veterans' experience and related health issues.

## 4 DISCUSSION

We believe that this is the first published example of patient engagement in setting the research agenda for cancer screening, although a United Kingdom colorectal cancer research priority‐setting exercise did identify the need for optimizing screening practices.^32^ The research questions prioritized by the Veterans who participated in this project illustrate the importance of engaging Veterans in setting the research agenda. They emphasized the importance of patient uptake of an intervention (e.g., identifying outreach strategies) and its impact on quality of life (e.g., how screening results impact psychological health), rather than technical aspects like identifying high‐risk subsets or describing predictors of uptake, which are heavily reflected in the current literature. Our finding indicated that patients and researchers/doctors may have very different perspectives when it comes to illness. This is consistent with published comparisons of participant priorities for research with the research that is actually performed.^33^, ^34^, ^35^, ^36^ For example, for health professionals, their lens was usually narrowly focused on the disease itself and treatments. For patients, their wide‐angle lens took in the whole of their lives, of which disease was one small part. The challenge ahead for patient‐centred care is helping providers understand that other parts of patients' life (i.e., posttraumatic stress disorder or psychological health for Veterans) may play a bigger role in leading up to the current situation (i.e., chronic diseases, medical non‐adherence, higher mortality rates). Only by overcoming this challenge will patients and providers each be able to adjust the angles of their respective lenses so that their vision can come into common focus.^36^ Our finding highlights the importance of having patient representation for setting up research agenda. Without patient representation, research agendas would not align with information that patients deem as most important for them.^10^

Over the last 15 years, the number of publications describing the results of involving patients in research priority setting has multiplied. They have described a range of approaches from focus groups, to on‐line surveys, to complex multi‐step processes. While it is not clear what elements are most important to achieving actionable, patient‐centred insights, our experience suggests several considerations. First, by focusing on a relatively narrow clinical issue, our PAC identified questions that clearly indicated their priorities. Second, we believe that facilitated interaction of the Veterans with other stakeholders who suggested potential clinical questions made the Veterans aware of issues that may not have arisen in their personal experience. To mitigate the power imbalance between SG and our PAC, the project team met separately and repeatedly with the two groups before bringing them together. Moreover, the PAC met independently to select the topic areas that were most important and to rank the research questions, with SG input available as a resource. The PAC also requested education regarding the process of LDCT, since relatively few had experienced it. As a result, we arranged an interactive role play between a PCA member and a physician to demonstrate the patient–doctor communication regarding LDCT screening and consultation for PAC members. PAC members found it helpful because this increased their familiarity with the subject matter, decreasing dependence on SG expertise. Not having sufficient knowledge or personal experience for a specific topic is also one of the known challenges of co‐creation/coproduction during the stakeholder engagement process.^37^ We believe that creating user‐friendly activities to enhance patients' understanding of a specific medical topic is vital for PAC members to have sufficient knowledge to identify research priorities. Third, PAC members committed to serving as a resource for cancer screening research more broadly, or research in other areas of importance to Veterans outside this project. We believe that this commitment to ongoing participation made PAC members realize the importance attached to their input.

Lung cancer screening provides a unique opportunity to examine the uptake of a proven screening modality^38^, ^39^ when multiple factors complicate the implementation process. First, the National Lung Screening Trial,^40^ which demonstrated a disease‐specific survival benefit, was restricted to a subset of the population that might benefit. Second, even in this population, LDCT screening led to a range of adverse outcomes as well as survival benefits. Third, the cost per lung cancer death avoided varies widely across risk groups.^41^

This study contributes to the literature on patient engagement in several ways. First, it provides a detailed process of how to engage patients and stakeholders to identify research questions and set research priorities for at‐risk Veterans.^42^, ^43^ Second, it details an innovative, participatory patient‐centred design^44^ approach for designing engagement meetings. Our patient‐centred approach facilitates the co‐learning process (one of the PCORI Principles of patient and stakeholder engagement) between patients and healthcare providers and helps balance the power among stakeholders.^25^, ^26^ The PCD emphasizes both (1) ‘solving the right problem’ and (2) ‘doing so in a way that meets human needs and capabilities.’^45^ Third, we apply key patient/stakeholder engagement principles and best practices from the PCORI.^28^

We acknowledge that our approach to involving patients in research prioritization has some limitations. First, while the small group facilitated the development of trust, it may also have limited the range of insights. Ideally, bringing together a large number of patients and collecting their inputs via different modalities (i.e., online survey) such as methods proposed by the James Lind Alliance (JLA) for priority setting^46^ may enrich our understanding of patient priorities. Due to the limited scope and time constraints of the patient engagement grant that we received, we were not able to survey a large number of patients. As a result, we cannot perform formal statistical analyses on the voting outcomes beyond reporting patterns. However, this study demonstrated a detailed process of co‐learning and partnership dynamics. Specifically, we provided a carefully designed workflow of developing a research agenda through meetings among different stakeholders. Second, our focus on a limited clinical area, while allowing for more focused research questions, does not allow one to use this method to compare priorities in this area to other aspects of lung cancer care or to screening for other forms of cancer. Third, while we ensured that the PAC made decisions independent of the stakeholders who advised them, we cannot be sure if endorsement by respected professionals caused some questions to be assigned more weight than a purely patient‐driven approach. However, the PAC saved their highest rankings for questions that had not been suggested by the stakeholders. Thus, since the objective of the study was to generate *informed* patient‐centred research questions and priorities, we believe that this approach achieves an appropriate balance.

We anticipate that the next steps are twofold. First, policy makers, practitioners and decision‐makers within local VA and healthcare systems need to be informed about our findings. The research team has already started the dissemination process. For example, our findings were disseminated to the statewide Veterans service organizations. Second, final priorities will be disseminated with researchers so that they can utilize this information to guide the design of future research projects. In fact, our PAC members already provided additional consultation to several VA researchers outside the study. Together, we plan to investigate many of the priorities outlined here with large comparative effectiveness research studies.

Patient engagement has the potential to enrich our understanding of patient priorities for research. Given the current focus on developing patient‐centred research questions, we suggest future studies to evaluate various patient engagement approaches and determine what approach works best, under which circumstances. Successful approaches will build trust in the patient–researcher partnership, ensure that patients are meaningfully engaged throughout the process and include diverse patient experiences and perspectives. We present our patient engagement approach as one option for groups interested in facilitating collaboration among multiple stakeholders to identify shared research priorities. The study highlights a hypothetical counterfactual—how different would research priorities be if selected solely by scientists versus this diverse patient advisory council. We noted that the research priorities were not the same as those initially identified by the scientists/health professionals, which points to the outcome of this hypothetical counterfactual. This study calls for future studies to assess whether lung cancer screening actually becomes better optimized by targeting research priorities identified by scientists or priorities identified by patients through a patient‐centred engagement process. While we likely will not truly know the answer to that experiment, the current study does provide valuable guidance around how to conduct a high‐quality patient advisory council.

## CONFLICT OF INTERESTS

The authors declare that there are no conflict of interests.

## AUTHOR CONTRIBUTIONS

Onur Asan, Alice Yan and Jeff Whittle conceived the study and obtained the funding. Alice Yan, Katinka Hooyer and Jeff Whittle wrote the manuscript with input from all authors. Alice Yan, Katinka Hooyer, Mark Flower and Jeff Whittle contributed to the implementations of the project. Alice Yan and Jeff Whittle contributed to the revision of the manuscript. Jeff Whittle helped supervise the project. All authors were involved in critical revision of the article. All authors discussed the results and contributed to the final manuscript.

## Data Availability

The data used to support the findings of this study are available from the corresponding author upon request.
